# Perilla Fruit Water Extract Attenuates Inflammatory Responses and Alleviates Neutrophil Recruitment via MAPK/JNK-AP-1/c-Fos Signaling Pathway in ARDS Animal Model

**DOI:** 10.1155/2022/4444513

**Published:** 2022-06-30

**Authors:** Nai-Chun Ting, Yu-Hao Chen, Jeng-Chang Chen, Wen-Chung Huang, Chian-Jiun Liou, Li-Chen Chen, Sien-Hung Yang, Ming-Ling Kuo

**Affiliations:** ^1^Graduate Institute of Clinical Medical Sciences, College of Medicine, Chang Gung University, Taoyuan, Taiwan; ^2^Department of Microbiology and Immunology, Graduate Institute of Biomedical Sciences, Chang Gung University, Taoyuan, Taiwan; ^3^Department of Surgery, Chang Gung Memorial Hospital-Linko, College of Medicine, Chang Gung University, Taoyuan, Taiwan; ^4^Department of Medicine, College of Medicine, Chang Gung University, Taoyuan, Taiwan; ^5^Graduate Institute of Health Industry Technology Research Center for Food and Cosmetic Safety, Chang Gung University of Science and Technology, Taoyuan, Taiwan; ^6^Research Center for Chinese Herbal Medicine College of Human Ecology, Chang Gung University of Science and Technology, Taoyuan, Taiwan; ^7^Division of Allergy, Asthma, and Rheumatology Department of Pediatrics, Chang Gung Memorial Hospital, Taoyuan, Taiwan; ^8^Department of Pediatrics, New Taipei Municipal Tucheng Hospital, New Taipei, Taiwan; ^9^School of Traditional Chinese Medicine, College of Medicine, Chang Gung University, Taoyuan, Taiwan; ^10^Division of Chinese Internal Medicine Center for Traditional Chinese Medicine Chang Gung Memorial Hospital, Taoyuan, Taiwan

## Abstract

Airway respiratory distress syndrome (ARDS) is usually caused by a severe pulmonary infection. However, there is currently no effective treatment for ARDS. Traditional Chinese medicine (TCM) has been shown to effectively treat inflammatory lung diseases, but a clear mechanism of action of TCM is not available. Perilla fruit water extract (PFWE) has been used to treat cough, excessive mucus production, and some pulmonary diseases. Thus, we propose that PFWE may be able to reduce lung inflammation and neutrophil infiltration in a lipopolysaccharide (LPS)-stimulated murine model. C57BL/6 mice were stimulated with LPS (10 *μ*g/mouse) by intratracheal (IT) injection and treated with three doses of PFWE (2, 5, and 8 g/kg) by intraperitoneal (IP) injections. To investigate possible mechanisms, A549 cells were treated with PFWE and stimulated with LPS. Our results showed that PFWE decreased airway resistance, neutrophil infiltration, vessel permeability, and interleukin (IL)-6 and chemokine (C-C motif) ligand 2 (CCL2/MCP-1) expressions *in vivo*. In addition, the PFWE inhibited the expression of IL-6, CCL2/MCP-1, chemokine (CXC motif) ligand 1 (CXCL1/GRO*α*), and IL-8 *in vitro*. Moreover, PFWE also inhibited the MAPK/JNK-AP-1/c-Fos signaling pathway in A549 cells. In conclusion, we demonstrated that PFWE attenuated pro-inflammatory cytokine and chemokine levels and downregulated neutrophil recruitment through the MAPK/JNK-AP-1/c-Fos pathway. Thus, PFWE can be a potential drug to assist the treatment of ARDS.

## 1. Introduction

Most patients with airway respiratory distress syndrome (ARDS) usually exhibit symptoms, such as hypoxemia, vessel hyperpermeability, neutrophil infiltration, edema, and shortness of breath [1, 2]. Based on patient-level meta-analysis, respiratory symptom severity, lung X-ray bilateral opacity detection, pulmonary edema detection, and oxygenation reduction, the Berlin definition of ARDS was developed to evaluate the ability of the stages of mild, moderate, and severe ARDS in predicting mortality and median duration of mechanical ventilation in survivors [1]. Pulmonary infection by bacteria and viruses is the most common pathogenic factor leading to ARDS with high morbidity and mortality rates. In the United States, approximately 70,000 people died of ARDS every year, and the morbidity has gradually increased in Taiwan from 1997 to 2011 [2–4]. In addition to the damage caused by infectious pathogens, the affected lungs suffer from host responses to molecules of pathogen-associated molecular patterns (PAMPs) or damage-associated molecular patterns (DAMPs), which cause a reduction in gas exchange function. The increased permeability of endothelial and epithelial cells leads to excessive fluid accumulation in the interstitium and alveolar spaces. Consequently, the lymph vessels and pulmonary circulation are unable to assist in fluid efflux, leading to edema and alveolar dysfunction. In addition, lung epithelial cells secrete pro-inflammatory cytokines such as interleukin (IL)-1, IL-6, and tumor necrosis factor (TNF)-*α* [5, 6]. In addition, the affected epithelial cells also secrete excessive chemokines, including chemokine (CXC motif) ligand 1 (CXCL1/GRO-*α*), CXCL2 (MIP-2), and chemokine (C-C motif) ligand 2 (CCL2/MCP-1), which bind C-X-C chemokine receptor type 2 (CXCR2) or C-C chemokine receptor 2 (CCR2), and then, the neutrophils were attracted into lung tissue by chemokines [7–9].

Lipopolysaccharide (LPS) is an endotoxin present on the walls of gram-negative bacteria. LPS is a prominent stimulus that induces leukocyte accumulation, lung edema, and inflammatory cytokine production in many pneumonia models [10, 11]. LPS activates the downstream Toll-like receptor 4 (TLR4) signaling pathway, followed by the activation of mitogen-activated protein kinases (MAPKs), including ERK, JNK, and p38 [12]. The key transcription factor for chemokine expression, activator protein 1 (AP-1), is then activated [13, 14].

Perilla fruit (PF) is the seed of *Perilla frutescens* (L.) Britton, belonging to the Labiatae family. PF, a traditional Chinese medicine (TCM) herb, is commonly used to treat cough and sputum. In addition, the leaves and stems have different therapeutic applications in TCM [15]. Although many studies have shown that the leaves and stems of *P. frutescens* have anti-inflammatory and antioxidative effects [16, 17], few studies have discussed the mechanisms of PF in pulmonary diseases. The aim of this study was to investigate the potential of PF in the treatment of lung infections and define the possible mechanisms.

## 2. Materials and Methods

### 2.1. The Preparation of Perilla Fruit Water Extract (PFWE) and High-Performance Liquid Chromatography (HPLC) Analysis

The PFWE was prepared and gifted by the Sheng Chang Pharmaceutical Company (Taoyuan, Taiwan). Briefly, seeds of *P. frutescens* were soaked in water (1 : 20) before boiling for 30 minutes. The extract was collected via centrifugation and evaporated under vacuum. The doses used in the following experiments were converted based on the dry weight of the PFWE. The HPLC profiles of PFWE were detected using Hitachi HPLC (L-2000 series, Chiyoda, Tokyo Japan) with a reverse-phase column (COSMOSIL 15C18) and rosmarinic acid as the indicating compounds (Figure S1).

### 2.2. LPS-Induced ARDS Murine Model and PFWE Treatment

Eight-week-old male C57BL/6 mice were purchased from the National Laboratory Animal Center (NLAC) in Taiwan. The animal experiments were approved by the Institutional Animal Care and Use Committee in Chang Gung University (CGU108-211) and Chang Gung University of Science and Technology (CGUST 2019-013). The mice were maintained in the research animal vivarium in Chang Gung University under the Guidelines for the Care and Use of Laboratory Animals. The mice were divided into five groups (normal, LPS, and three treatment groups: 2, 5, and 8 g/kg). Each mouse received an intratracheal (IT) injection of LPS (10 *μ*g, Merck, Burlington, MA, USA) on day 0 to mimic infection by gram-negative bacteria. For the PFWE-treated groups, the mice were administered different doses of PFWE by intraperitoneal (IP) injections at 1 h, one day, and two days after the LPS injection. The mice in the normal group were injected with normal saline instead of LPS and were not treated with PFWE. The mice were sacrificed by cervical dislocation, while they were anaesthetized with isoflurane (Piramal Critical Care, PA, USA) on day three.

### 2.3. Lung Cell Preparation and Flow Cytometry

Lung tissues were collected and subjected to collagenase digestion (200 U/ml collagenase type I, Worthington Biochemical Corporation, Lakewood, NJ, USA) in Roswell Park Memorial institute 1640 (RPMI) medium containing 1% penicillin/streptomycin, 0.05% DNase I (all from Thermo Fisher Scientific, Waltham, MA, USA), and 1% HEPES (Merck) at 37°C for 1 h. The reaction was stopped using a complete RPMI medium containing 5% fetal bovine serum (FBS, Thermo Fisher Scientific). The single suspended cells were stained with eFluor 506 antimouse CD45 antibody (Thermo Fisher Scientific), Ghost dye™ Red 780 viability Dye (TONBO Biosciences, San Diego, CA, USA), and PE antimouse Ly-6G/Ly-6C (Gr-1) antibody (Biolegend, San Diego, CA, USA) to detect the neutrophil population and then analyzed by flow cytometry (Attune NxT Flow Cytometer, Thermo Fisher Scientific).

### 2.4. Histological Analysis

Mouse lung tissues were removed and fixed in 4% formaldehyde. Tissue sections were stained with hematoxylin and eosin (H&E) to detect inflammatory cell infiltration. Digital images were acquired at 200× magnification using an Olympus microscope (Olympus, Shinjuku, Tokyo, Japan) and quantified using Image J software (developed by the National Institutes of Health, Bethesda, MD, USA).

### 2.5. Vessel Permeability

Lung vessel permeability was determined using Evans blue staining (Merck). The mice received an intravenous (IV) injection of Evans blue (0.5% in 200 *μ*l) after anesthesia. Thirty minutes later, cardiac perfusion was performed to remove the blood, and the lungs were harvested for image acquisition. The lung tissues were also dried at 65°C overnight, and Evans blue in each tissue sample was dissolved by incubation with formamide (Merck) at 55°C for another 24 h. The supernatants were collected, and the amount of Evans blue was detected using a microplate reader (Multiskan FC, Thermo Fisher Scientific) at an absorbance of 610 nm.

### 2.6. Bronchoalveolar Lavage Fluid (BALF) Collection and Leukocyte Counting

After the mice were sacrificed, the trachea was intubated using an indwelling needle (BD Infusion Therapy System Inc., Franklin Lake, NJ, USA) to wash the lungs and airways with 3 ml normal saline. The supernatant of the first 1 ml was centrifuged, and the fluid was collected and stored at −80°C for cytokine and chemokine detection. Cells in the first and the other 2 ml were harvested, fixed on slides, and stained with Wright Giemsa stain (Merck). Approximately, 200 cells were counted and the percentage of neutrophils and total cell numbers were calculated.

### 2.7. Cell Line and Cell Culture

The human lung epithelial cell line, A549, was obtained from the Bioresource Collection and Research Center of the Food Industry Research and Development Institute (FIRDI; Hsin-Chu, Taiwan). The cells were cultured in Ham's F-12K (Kaighn's) medium supplemented with 10% FBS, 1% penicillin/streptomycin, and 1% L-glutamine (all from Thermo Fisher Scientific). Cells (2 × 10^5^/well) were seeded for 24 h. The cells were then treated with PFWE (50, 200, and 800 *μ*g/ml) for 18 h and LPS (1 *μ*g/ml) for 6 or 24 h for cytokine or chemokine analyses of the supernatants and RNA extraction. In order to detect the phosphorylated levels of the proteins in the signaling pathways by western blotting, the cells were harvested 30 min or 1 h after LPS treatment.

### 2.8. Cell Viability Assay

Cell viability was determined using the cell counting kit-8 (CCK-8, Merck). A549 cells (2 × 10^5^/well) were treated with different doses of PFWE for 18 h, followed by LPS stimulation (1 *μ*g/ml) for another 24 h. The cells were then incubated with CCK-8 solution at 37°C for 1 h. Cell viability was determined by measuring the absorbance at 450 nm using a microplate reader (Multiskan FC, Thermo Fisher Scientific).

### 2.9. Enzyme-Linked Immunosorbent Assay (ELISA)

BALF and A549 culture supernatants were collected and subjected to cytokine or chemokine detection. The ELISA kits were used to determine the levels of IL-6, CXCL1/GRO*α*, CCL2/MCP-1, and IL-8 (R&D Systems, Minneapolis, MN, USA). The concentration of each cytokine or chemokine was calculated based on the standard curve that was constructed with recombinant proteins and measurement of absorbance at 450 nm by a microplate reader (Multiskan FC, Thermo Fisher Scientific).

### 2.10. RNA Extraction and Quantitative Real-Time PCR Analysis

RNA was extracted from the lung tissues and A549 cells using TRIzol reagent (Biotools, New Taipei, Taiwan). The MMLV Reverse Transcription Kit (PROTECH, Taipei, Taiwan) was used to synthesize the cDNA. Quantitative real-time PCR (q-PCR) was performed with iQ™ SYBR Green Supermix (Bio-Red, Hercules, CA, USA) using a spectrofluorometric thermal cycler (Roche, Basel, Switzerland). The specific primers used for each gene are listed in Table 1.

### 2.11. Western Blot Assay

The cells were lysed with a buffer containing radioimmunoprecipitation assay (RIPA) (Visual Protein, Taipei, Taiwan), protease inhibitor (Bionovax, Taoyuan, Taiwan), and phosphate inhibitor (MedChem Express, Monmouth Junction, NJ, USA) and then separated on 10% sodium dodecyl phosphate-polyacrylamide gel electrophoresis (SDS-PAGE) gels. The gels were transferred to 0.45 *μ*m nitrocellulose blotting membranes (GE Healthcare, Chicago, IL, USA) and incubated with primary antibodies specific to each protein in the signaling pathway at 4°C overnight. The membranes were incubated with secondary antibodies at room temperature for 1 h and developed with luminol/enhancer solution (Merck) to obtain specific protein signals using the BioSpectrum 600 System (UVP, Upland, California, USA). Primary antibodies included JNK, phospho-JNK, c-Fos, phospho-c-Fos, and *β*-actin (Cell Signaling Technology, Danvers, MA, USA).

### 2.12. Statistical Analysis

The significance of all data was analyzed using the Prism 9 software (GraphPad Software, Inc., San Diego, CA, USA). Data are expressed as mean ± standard error of mean (SEM) of at least three independent experiments were evaluated by one-way analysis of variance (ANOVA) followed by the Kruskal–Wallis test or Student's *t*-test (nonparametric tests) with the Mann–Whitney test. Significance was set at ^*∗*^*p* < 0.05, ^*∗∗*^*p* < 0.01, and ^*∗∗∗*^*p* < 0.001.

## 3. Results

### 3.1. PFWE Decreased Airway Resistance and Neutrophil Infiltration

**Figure 1:**
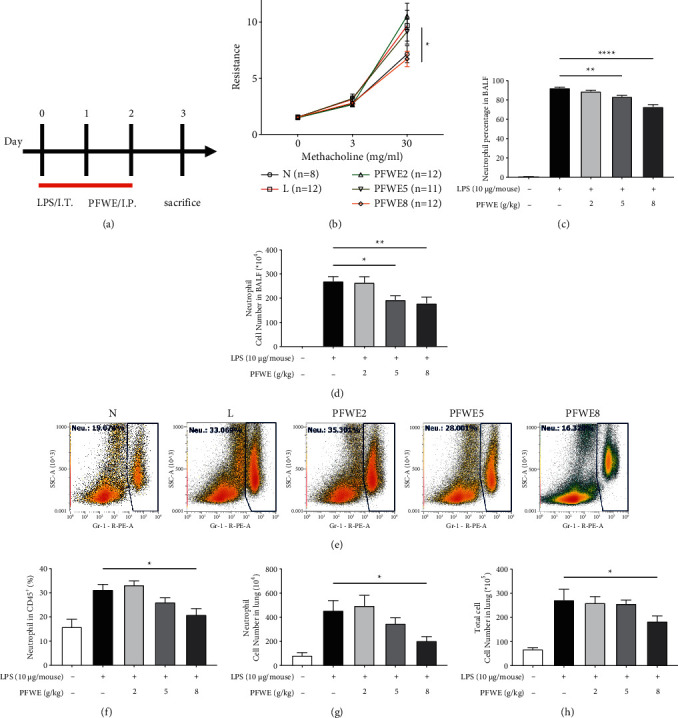
PFWE reduced airway resistance and neutrophil infiltration in ARDS model mice. (a) The mice received LPS 10 *μ*g/mouse by intratracheal (IT) injection and were treated with different doses of PFWE by intraperitoneal (IP) administration on days 0–2. The mice were divided into five groups: normal (N), LPS-stimulated (L), and three treatment groups (PFWE 2, 5, and 8 g/kg). (b) Airway resistance was determined with the stimulation of different doses of methacholine (0, 3, and 30 mg/ml) (*n* = 8−12 mice in each group). The (c) percentage and (d) cell number of neutrophils in BALF of mice in each group (*n* = 10 in each group). (e) The representative histograms of Gr-1^+^ cells are shown. (f) The percentages of Gr-1^+^ cells in CD45^+^ cells, (g) Gr-1^+^ cell number, and (h) total leukocytes in the lungs were examined by flow cytometry (*n* = 8 in each group). Data are presented as mean ± SEM (^*∗*^*p* < 0.05, ^*∗∗*^*p* < 0.01, and ^*∗∗∗∗*^*p* < 0.0001) compared with the LPS-stimulated group and analyzed using one-way analysis of variance (ANOVA) with the Kruskal–Wallis test.

We used the LPS-induced ARDS mouse model to assess the anti-inflammatory effects of PFWE (Figure 1(a)). First, we determined the changes in airway resistance based on methacholine challenges to promote bronchoconstriction with or without the treatment at different doses (0, 3, and 30 mg/ml). The results showed that the treatment with 8 g/kg PFWE alleviated airway resistance (Figure 1(b)). In addition, the percentages and cell numbers of neutrophils were also significantly decreased in the BALF of mice treated with 5 or 8 g/kg PFWE (Figures 1(c) and 1(d), respectively). In addition to neutrophil infiltration in the airways, cell populations in the lung tissues were subjected to flow cytometry using cell lineage-specific antibodies. The Gr-1 on the cell surface is a typical neutrophil marker. The results indicated a decrease in Gr-1^+^ cell population among CD45^+^ cells in the lungs of the PFWE-treated mice (Figure 1(e)). The quantitative data confirmed that the percentages (Figure 1(f)) and cell numbers (Figure 1(g)) of neutrophils and total leukocytes (Figure 1(h)) were significantly reduced in the lung lysates of PFWE-treated mice.

### 3.2. PFWE Suppressed Lung Inflammation and Vessel Permeability

**Figure 2:**
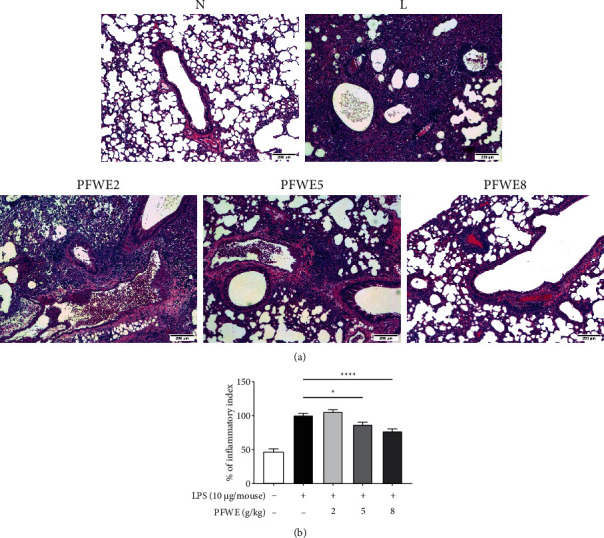
PFWE decreased neutrophil infiltration in ARDS model mice. (a) The tissue slides were stained with hematoxylin and eosin stain (H&E) and were observed at 200× magnification. (b) The quantitative data are shown. The tissue slides were photographed by a microscope and quantitated using Image J software. Mice groups were divided, as shown in Figure 1. The data are presented as mean ± SEM (^*∗*^*p* < 0.05 and ^*∗∗∗∗*^*p* < 0.0001) compared with the LPS-stimulated group and analyzed using one-way analysis of variance (ANOVA) with the Kruskal–Wallis test.

**Figure 3:**
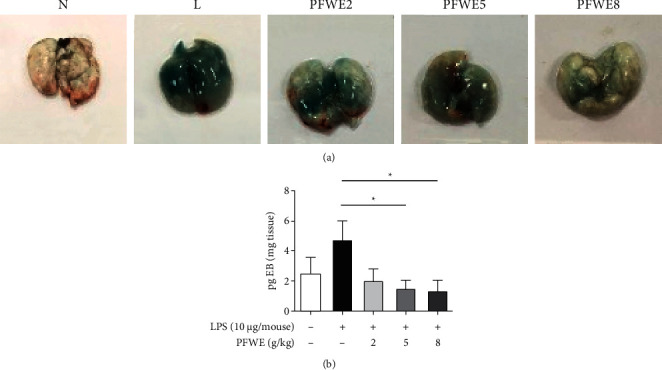
PFWE decreased vessel permeability that was induced by LPS stimulation. (a) The vessel permeability was observed by Evans blue dye staining. (b) The quantitative data are shown. Mice groups were divided, as shown in Figure 1. The data were analyzed, as shown in Figure 2.

The effect of PFWE on lung inflammation was also examined by H&E staining of tissue sections (Figure 2(a)). The quantified data showed a significant decrease in cell infiltration in the lungs of mice treated with 5 or 8 g/kg (Figure 2(b)). Since LPS can enhance vessel permeability in the lungs, we also examined whether PFWE protects against vessel leakage. Evans blue was used to determine the vessel permeability, due to its binding capacity to albumin in the affected tissues. The results showed that PFWE decreased the dye deposition caused by LPS (Figure 3(a)). The quantified data showed a significant reduction in Evans blue infiltration into the lungs of mice treated with 5 or 8 g/kg PFWE (Figure 3(b)).

### 3.3. PFWE Decreased the Level of Cytokines and Chemokines in ARDS Model Mice

**Figure 4:**
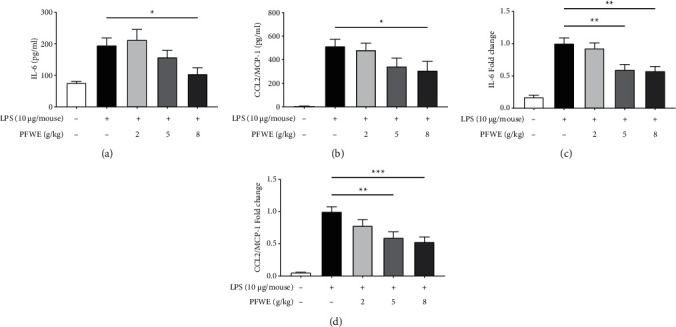
PFWE decreased the expression of cytokines and chemokines in the lungs. The levels of (a) IL-6 and (b) CCL2/MCP-1 were detected by ELISA in the BALF (*n* = 8–10 in each group). The gene expressions of (c) IL-6 and (d) CCL2/MCP-1 were determined by q-PCR in the lungs (*n* = 10 in each group). Mice groups were divided, as shown in Figure 1. The data are presented as mean ± SEM (^*∗*^*p* < 0.05 and ^*∗∗*^*p* < 0.01) compared with the LPS-stimulated group and analyzed using one-way analysis of variance (ANOVA) with the Kruskal–Wallis test.

Next, we investigated whether PFWE could affect cytokine and chemokine levels in the LPS-induced inflamed lungs. The data indicated that the treatment with 8 g/kg of PFWE significantly reduced the concentrations of IL-6 (a pro-inflammatory cytokine) and CCL2/MCP-1 (a chemokine) in BALF (Figures 4(a) and 4(b), respectively). The RNA expression of IL-6 and CCL2/MCP-1 genes in the lung tissues was also examined. The results showed that both doses of 5 and 8 g/kg PFWE efficiently suppressed the expression of IL-6 and CCL2/MCP-1 genes (Figures 4(c) and 4(d), respectively). However, the BALF levels or gene expressions of other pro-inflammatory cytokines or chemokines, such as IL-1*β*, TNF-*α*, or CXCL1/KC were not affected by PFWE treatment (data not shown).

### 3.4. PFWE Inhibited the Production of Cytokines and Chemokines in LPS-Activated A549 Cells

**Figure 5:**
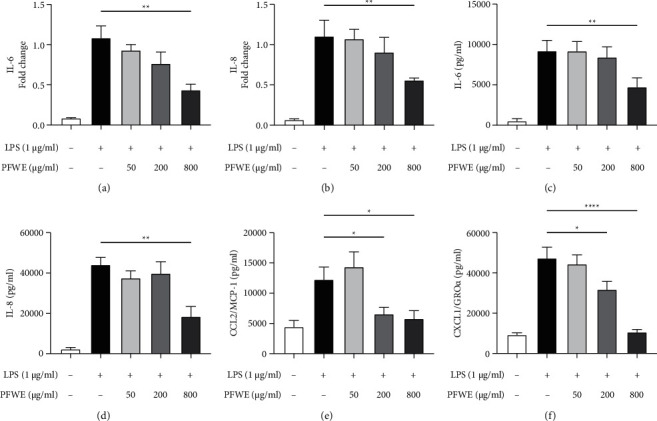
PFWE inhibited the expression of pro-inflammatory cytokines and chemokines in A549 cells. The cells were stimulated with LPS (1 *μ*g/ml) and treated with or without different doses of PFWE for 6 h The RNA expression of (a) IL-6 and (b) IL-8 was examined by q-PCR (*n* = 8 in each group). The levels of (c) IL-6, (d) IL-8, (e) CCL2/MCP-1, and (f) CXCL1/GRO*α* in the supernatants of A549 cells stimulated with LPS for 24 h were determined (*n* = 12 in each group). The data are presented as mean ± SEM (^*∗*^*p* < 0.05, ^*∗∗*^*p* < 0.01, and ^*∗∗∗∗*^*p* < 0.0001) compared with the LPS-stimulated group and analyzed using one-way analysis of variance (ANOVA) with the Kruskal–Wallis test.

Because the epithelium is the first line of defense against bacterial infection and we found that PFWE inhibited the expression of IL-6 and CCL2/MCP-1 genes in the LPS-challenged lungs in mice, we further investigated the potential mechanisms. Here, LPS-stimulated human lung epithelial A549 cells were used as an *in vitro* assay model. The results showed that the gene expression of IL-6 and IL-8 was significantly suppressed in LPS-stimulated A549 cells cultured in 800 *μ*g/ml PFWE for 6 h (Figures 5(a) and 5(b)). The reduction in the levels of cytokines such as IL-6 and IL-8 was also detected in the 24 h-culture supernatants of A549 cells (Figures 5(c) and 5(d)). In addition to IL-8, which is a key factor in chemoattractant human neutrophils, we also examined the levels of other neutrophil-specific chemokines, CCL2/MCP-1 and CXCL1/GRO*α*. The results indicate that the levels of these chemokines were also significantly reduced in the cultures with PFWE at 200 and 800 *μ*g/ml (CCL2/MCP-1 (Figure 5(e)) and CXCL1/GRO*α* (Figure 5(f))). The reduction in cytokine or chemokine production was not due to the direct cytotoxic effect of PFWE, since the cell viability was not affected by culturing with different doses of PFWE (50, 200, or 800 *μ*g/ml) for 24 h (Figure S2). Thus, PFWE is able to inhibit the production of cytokines and chemokines in LPS-activated A549 cells.

### 3.5. PFWE Inhibits the JNK-AP-1 Signaling Pathway in LPS-Stimulated A549 Cells

**Figure 6:**
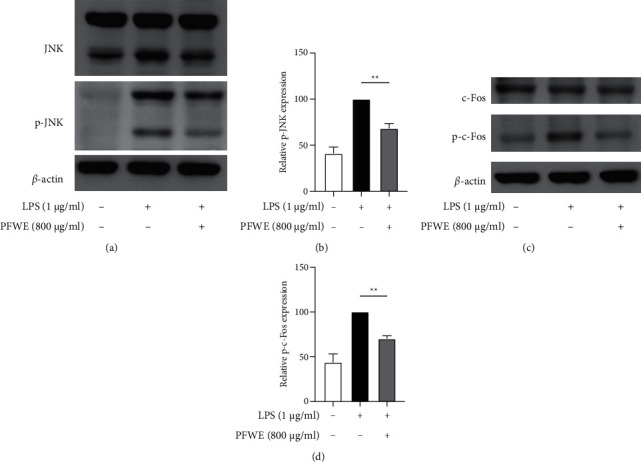
PFWE inhibited the MAPK/JNK-c-Fos signaling pathway. The LPS-stimulated A549 cells were treated without or with PFWE (800 *μ*g/ml). (a) and (c) The JNK, phosphorylated-JNK (p-JNK), c-Fos, and the phosphorylated-c-Fos (p-c-Fos) were detected by western blotting. (b) and (d) The quantitative results of p-JNK or p-c-Fos were calculated based on the normalization of the signals to the signals of JNK or c-Fos, respectively (*n* = 6 in each group). The data are presented as mean ± SEM (^*∗∗*^*p* < 0.01) compared with the LPS-stimulated group and analyzed using the *t*-test (nonparametric tests) and the Mann–Whitney test.

Next, we investigated the possible signaling pathways affected by PFWE in LPS-stimulated A549 epithelial cells. We found that the phosphorylation of JNK was significantly inhibited by the treatment with 800 *μ*g/ml PFWE for 30 minutes in the LPS-stimulated A549 cells (Figures 6(a) and 6(b)). We also examined the activity of AP-1, the main component of the JNK downstream transcription factor. The data indicated that PFWE significantly reduced phosphorylated c-Fos levels following LPS stimulation for 60 minutes (Figures 6(c) and 6(d)). However, the downstream molecules of the TLR4 signaling pathway, such as NF-*κ*B, ERK, and p38, or c-Jun, were not affected by PFWE at different time points (data not shown).

## 4. Discussion

ARDS is usually caused by pneumonia, sepsis, trauma, and other factors [18]. Most therapeutic strategies focus on supportive care to administer surfactants, steroids, or antibiotics [18]. In recent years, many biologics targeting IL-6 receptor have been developed to control ARDS, including tocilizumab, satralizumab, and sarilumab, and have shown acceptable efficacy. However, some severe adverse effects regarding the susceptibility to infection and others dampened the use of these biologics [19, 20]. In addition, biologics are usually expensive and not everyone can afford them. On the other hand, TCM has been used to treat many diseases for more than thousands of years, although a clear mechanism is usually not available.

Our data indicated that PFWE was able to reduce the population of inflammatory cells, particularly neutrophils, in both BALF and lung lysates. PFWE also reduced the permeability of LPS-challenged lungs. In addition, PFWE decreased the gene expression and protein production of inflammatory cytokine (IL-6) and chemokine (CCL2/MCP-1). Although this is the first study to show the efficacy of PFWE in controlling ARDS, similar results have been reported by applying other TCM, such as *Indigo naturalis*, Huang Qin, or *Sophorae flavescentis Radix* [21–23]. Our data further support the use of TCM for the treatment of pulmonary diseases in the acute phase. Based on a common method, we used high-temperature heating water to prepare the PFWE. High temperatures have the potential to increase the solubility of hydrophilic active phytochemicals, such as phenolic acids, flavonoids, and polysaccharides in the extracts. In particular, the extracts from fruit seeds usually require high temperatures to efficiently dissolve more phytochemicals in the solvent. Excess temperature and time may affect the activity of TCMs [24]. The active components in TCM are routinely measured using HPLC to confirm the quality of TCM to match the established standards in the pharmacopeia. In addition, the methods of high-temperature TCM extraction are commonly accepted by scientists worldwide for the studies in determining the drug responses and mechanisms of alternative medicines [25].

Epithelial cells, as the first line to be invaded by foreign pathogens, build a barrier to resist the infection [26]. When epithelial cells suffer from pathogenic stimuli, they secrete massive amounts of proinflammatory cytokines and chemokines to attract neutrophil migration. Thus, we used LPS-stimulated A549 cells as a lung epithelial cell model to validate the effects of PFWE in controlling the inflammatory response. Previous studies have demonstrated that CXCL1/GRO*α*, IL-8, and CCL2/MCP-1 regulated neutrophil recruitment upon pneumonia infection [27, 28]. In A549 cell cultures, we found that CXCL1/GRO*α*, IL-8, and CCL2/MCP-1 levels were significantly reduced by PFWE treatment. These data suggest that the suppression of the neutrophil infiltration suppressed by PFWE may be mediated by the downregulation of cytokines and chemokines.

PFWE also prevented the LPS-induced increase in lung permeability. LPS causes damage to alveolar epithelial cells and tight junctions (TJs), which are comprised of transmembrane proteins (occludins, claudins, and JAM), adhesion junctions (VE-cadherin), and zonula occludins (ZO) [2, 29]. Increased permeability is likely to contribute to neutrophil transmigration. Many signaling pathways regulate tight junctions, including protein kinases A, C, and G (PKA, PKC, and PKG), Rho kinases, myosin light chain kinase (MLCK), and MAPK [30, 31]. JNK activation causes epithelial barrier dysfunction, leading to increased permeability and damage to tight junctions by reducing the concentrations of ZO-1 and occludins [32]. SP600125, a JNK inhibitor, can prevent the loss of occludins in stretch-induced primary rat alveolar epithelial type two cells [33]. SP600125 also decreased IL-6 levels and upregulated claudin-4 expression in the LPS-stimulated ARDS animal model and A549 cell line [34]. Thus, PFWE might be able to repair tight junction proteins via suppression of the JNK signaling pathway in our ARDS model. The mechanism of action of *Houttuynia cordata* was similar to that of PFWE. Similarly, reduced inflammatory cell infiltration in BALF and lung tissues was observed following treatment with the *H. cordata* extract. The infiltrated polymorphonuclear leukocytes were detected mainly based on histological and IHC analyses [35]. Our current study further demonstrated the cell populations using flow cytometry. Nevertheless, *H. cordata* was also found to reduce lung permeability and increase the expression of ZO-1. Therefore, the effects of TCM on the integrity of tight junctions may be the focus of future research [36].

The activated TLR4 signaling pathway may induce the secretion of proinflammatory cytokines and chemokines secretion, such as IL-6, TNF-*α*, CCL2/MCP-1, CXCL1/GRO*α*, and IL-8 via MAPK signals [37]. Previous studies have also demonstrated that natural compounds alleviated LPS-induced ARDS by suppressing the activity of the MAPK pathway and controlling lung edema, cell infiltration, and permeability, as well as the expression of proinflammatory cytokines [38, 39]. In addition, hesperidin decreased the expression of proinflammatory cytokines, including TNF-*α*, IL-1*β*, IL-6 and enhanced that of anti-inflammatory cytokines, IL-10, IL-4, and IL-12 by JNK and p38 MAPK pathways [40]. Oxymatrine reduced neutrophil infiltration and inflammatory responses of LPS-induced acute lung injury animal model and suppressed the JNK signaling pathway [23]. In this study, we demonstrate that the PFWE downregulates MAPK/JNK-AP-1/c-Fos signaling without a significant effect on the NF-*κ*B signaling pathway to inhibit the levels of IL-6, IL-8, CXCL1/GRO*α*, and CCL2/MCP-1 in the supernatants, and IL-6 and IL-8 gene expressions in LPS-stimulated A549 cells.

The reduction of inflammatory responses has the potential to decrease epithelial injury and neutrophil recruitment to achieve a therapeutic effect. Currently, the severe acute respiratory syndrome coronavirus-2 (SARS-CoV 2) wreaks havoc worldwide and causes high morbidity and mortality. Some patients developed a severe disease stage with a cytokine storm, especially the IL-6 production, in their pulmonary tissues approximately one week after viral infection [41, 42]. Recently, some studies have indicated high levels of IL-6 production in patients with COVID-19 at the acute and final stages [43, 44]. However, Chinese medicine with a high efficacy for ARDS may be applied for COVID-19 treatment. For example, HuoxiangZhengqi (HXZQ), LianhuaQingwen (LHQW), ShufengJiedu (SFJD), and XueBijing (XBJ) have been shown to inhibit the levels of proinflammatory cytokines and block cytokine storm, modulate immune responses, and protect against multiple organ failure in the SARS-CoV 2-infected patients [45]. Since TCM has a high potential for application in lung injury treatment, we speculate that PFWE may be able to treat severe lung infection diseases.

In our study, we demonstrated that PFWE reduced neutrophil infiltration and inflammatory response in LPS-induced ARDS murine model. Most previous reports applied drug treatment a few hours or days prior to LPS stimulation [46, 47]. In this study, PFWE was administered after the LPS stimulation. Considering that TCMs usually require longer treatment times, we examined the efficacy of PFWE three days after LPS stimulation in our ARDS animal model. In this situation, some inflammatory indices may have declined to the levels, not easily showing significant reduction by PFWE treatment. In recent years, the relationship between TCM and the gut microbiome has been described. In addition, the gut microbiome can affect the TCM pharmacological reaction of TCM in the hosts [48]. Thus, the effect of PFWE may be different if it is administered orally which needs to be investigated further.

## 5. Conclusions

In conclusion, PFWE reduced neutrophil infiltration and IL-6 and CCL2/MCP-1 expressions *in vivo*. We further showed that PFWE inhibited IL-8, CXCL1/GRO*α*, and CCL2/MCP-1 expressions, as well as the MAPK/JNK-AP-1/c-Fos signaling pathway in LPS-stimulated A549 cells. Thus, we demonstrated that PFWE effectively attenuated inflammatory cytokine and chemokine levels and downregulated neutrophil recruitment through the MAPK/JNK-AP-1/c-Fos pathway.

## Figures and Tables

**Table 1:** Primer pairs used in q-PCR.

Gene	Forward (5′-3′)	Reverse (5′-3′)
Mouse		
IL-6	CCGGAGAGGAGACTTCACAG	TCCACGATTTCCCAGAGAAC
CCL2/MCP-1	TTAAAAACCTGGATCGGAACCAA	GCATTAGCTTCAGATTTACGGGT
*β*-actin	GGCTGTATTCCCCTCCATCG	CCAGTTGGTAACAATGCCATGT

Human		
IL-6	CCAATCTGGATTCAATGAGGAG	GGTCAGGGGTGGTTATTGCATC
IL-8	ACACTGCGCCAACACAGAAA	CAACCCTCTGCACCCAGTTT
GAPDH	GCAAATTCCATGGCACCG	TCGCCCCACTGATTTTGG

## Data Availability

The datasets used or analyzed during the current study are available from the corresponding author upon reasonable request.

## Supplementary Materials

Supplementary Materials Figure S1. HPLC profiles of seeds of *Perilla frutescens*; Figure S2. PFWE treatment did not alter the cell viability of A549 cells with or without LPS stimulation.
