# Supplementary material for: Perilla Fruit Water Extract Attenuates Inflammatory Responses and Alleviates Neutrophil Recruitment via MAPK/JNK-AP-1/c-Fos Signaling Pathway in ARDS Animal Model
Source: Evid Based Complement Alternat Med. 2022 Jun 30;2022:4444513. doi: 10.1155/2022/4444513 (PMC9262517; doi:10.1155/2022/4444513)
Supplement: Supplementary Materials — Figure S1. HPLC profiles of seeds of Perilla frutescens; Figure S2. PFWE treatment did not alter the cell viability of A549 cells with or without LPS stimulation. [file 4444513.f1.docx]

**Supplemental figures**


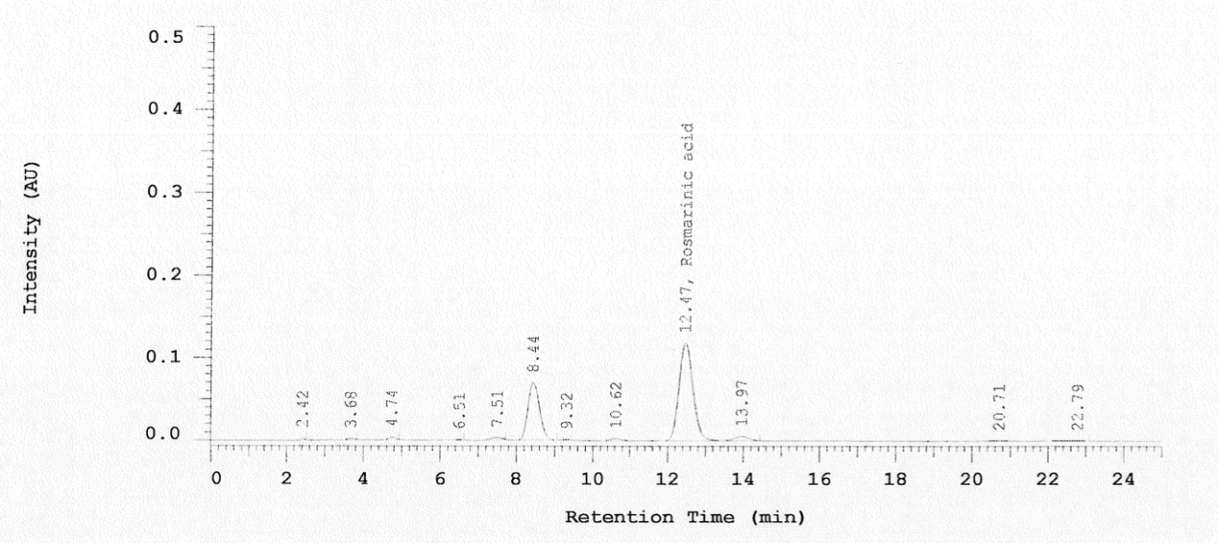

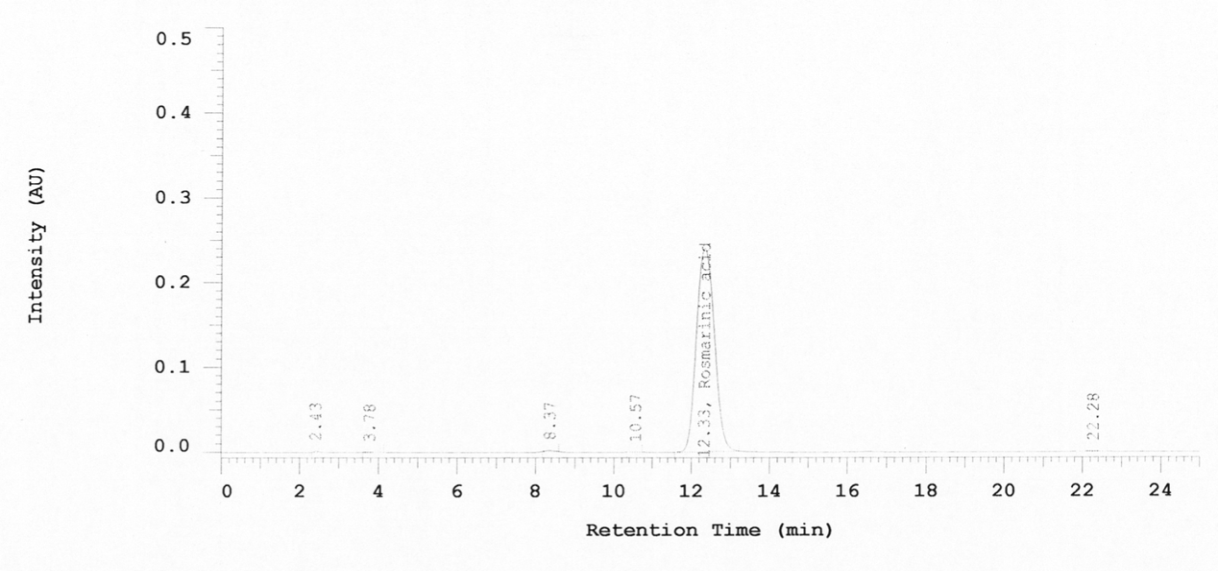


**(A)**

**(B)**

Figure. S1 HPLC profiles of seeds of perilla frutescens. The rosmarinic acid is considered to be an ingredient of traditional Chinese medicine for PFWE. (A) The peak of rosmarinic acid was shown at 12.45 min in PFWE. (B) The peak of the standard sample for rosmarinic acid was shown at 12.33 min.

Figure. S2 PFWE treatment did not alter the cell viability of A549 cells with or without LPS stimulation. The cells were treated with PFWE for 18 hours and stimulated with LPS for 24 hours. The viability was measured based on the CCK-8 test on the supernatant of each culture. Cells were divided into eight groups: cell only, LPS only, three doses of PFWE treatment without LPS, and three doses of PFWE treatment with LPS (n = 18 in each group).
